# Mutant *LRP6* Impairs Endothelial Cell Functions Associated with Familial Normolipidemic Coronary Artery Disease

**DOI:** 10.3390/ijms17071173

**Published:** 2016-07-22

**Authors:** Jian Guo, Yang Li, Yi-Hong Ren, Zhijun Sun, Jie Dong, Han Yan, Yujun Xu, Dao Wen Wang, Gu-Yan Zheng, Jie Du, Xiao-Li Tian

**Affiliations:** 1 Department of Human Population Genetics, Institute of Molecular Medicine, Peking University, Beijing 100871, China; deidei@163.com (J.G.); ly1306386063@pku.edu.cn (Y.L.); djqiu@163.com (J.D.); yanhan@pku.edu.cn (H.Y.); zhengguyan@163.com (G.-Y.Z.); 2 Department of Cardiovascular, PLA General Hospital, Beijing 100853, China; rainbowren301@163.com (Y.-H.R.); sunzj301@sohu.com (Z.S.); 3 The Institute of Hypertension and Department of Internal Medicine, Tongji Hospital, Tongji Medical College, Huazhong University of Science and Technology, Wuhan 430074, China; xuyujun3506@163.com (Y.X.); dwwang@tjh.tjmu.edu.cn (D.W.W.); 4 Beijing Anzhen Hospital, Capital Medical University, The Key Laboratory of Remodeling-Related Cardiovascular Diseases, Ministry of Education, Beijing Collaborative Innovation Center for Cardiovascular Disorders, Beijing Institute of Heart, Lung & Blood Vessel Disease, Beijing 100029, China; jiedubj@126.com; 5 Department of Human Population Genetics, Human Aging Research Institute and School of Life Science, Nanchang University, Nanchang 330031, China

**Keywords:** LDL receptor-related protein-6 (*LRP6*), normolipidemic, coronary artery disease, familial, endothelial cell dysfunction

## Abstract

Mutations in the genes low-density lipoprotein (LDL) receptor-related protein-6 (*LRP6*) and myocyte enhancer factor 2A (*MEF2A*) were reported in families with coronary artery disease (CAD). We intend to determine the mutational spectrum of these genes among hyperlipidemic and normolipidemic CAD families. Forty probands with early-onset CAD were recruited from 19 hyperlipidemic and 21 normolipidemic Chinese families. We sequenced all exons and intron-exon boundaries of *LRP6* and *MEF2A*, and found a novel heterozygous variant in *LRP6* from a proband with normolipidemic CAD. This variant led to a substitution of histidine to tyrosine (Y418H) in an evolutionarily conserved domain YWTD in exon 6 and was not found in 1025 unrelated healthy individuals. Co-segregated with CAD in the affected family, *LRP6_Y418H_* significantly debilitated the Wnt3a-associated signaling pathway, suppressed endothelial cell proliferation and migration, and decreased anti-apoptotic ability. However, it exhibited no influences on low-density lipoprotein cholesterol uptake. Thus, mutation Y418H in *LRP6* likely contributes to normolipidemic familial CAD via impairing endothelial cell functions and weakening the Wnt3a signaling pathway.

## 1. Introduction

Coronary artery disease (CAD), the most common cause of death, is characterized by the stenosis or occlusions of coronary arteries that are mostly caused by the progressive deposition of lipids and fibrous matrix (atherosclerotic plaques) in the arterial wall [1]. The key steps of atherosclerogenesis include dysfunction of the endothelium, lipoprotein deposition, recruitment of monocytes and lymphocytes, and proliferation of smooth muscle cells [2]. Endothelial cell survival, proliferation, and migration are critical to maintain the homeostasis and normal functions of endothelium [3].

The common form of CAD appears multi-factorial in etiology, involving an interaction between genetic and environmental factors [4,5]. Over the past decades, great efforts have been made to systematically search for genes or chromosomal loci associated with CAD at the genome level by family or population-based association studies, leading to the identification of numbers of susceptibility genes or loci [6,7,8,9,10]. In contrast, familial CAD is rare but highly penetrated, presenting a monogenic effect and Mendelian inheritance. Mutations in genes *MEF2A* and *LRP6* were previously identified as pathogenic or CAD-causing variants for familial CAD [11,12]. The detections of these loci or genes apparently increase our knowledge of understanding the molecular mechanism of CAD and may be helpful in improving the clinical treatment and drug discovery.

In addition to genetic influences, other risk factors for CAD are well established, such as elevated cholesterol, hypertension, obesity, and unhealthy lifestyles [13]. For example, hyperlipidemia, particularly the elevated level of low-density lipoprotein cholesterol (LDL-C), increases the risk for atherosclerosis which is fundamental to CAD [14,15,16]. In contrast, the high-density lipoprotein cholesterol (HDL-C) is inversely related with CAD [15,16,17]. Although hyperlipidemia is a significant risk factor for CAD, a considerable proportion of CAD patients are normolipidemic. It has been reported that only a third of CAD patients exhibit elevated cholesterol levels, especially LDL-C [18]. More than 40% and 60% of male CAD patients have normal ranges of serum LDL-C (<130 mg/dL, 3.4 mmol/L) and HDL-C levels (>35 mg/dL, 0.9 mmol/L) respectively [19]. Ultimately, how genetic determinants contribute differentially to hyperlipidemic and normolipidemic CAD remains largely unknown.

In this study, we recruited 40 Chinese Han families with early-onset hyperlipidemic or normolipidemic CAD, sequenced all exons and intron-exon boundaries of *LRP6* and *MEF2A*, two previously reported CAD genes, and characterized the functions of newly identified mutations.

## 2. Results

### 2.1. A Novel Mutation in LRP6 Was Identified in a Family with Normolipidemic CAD

**Figure 1:**
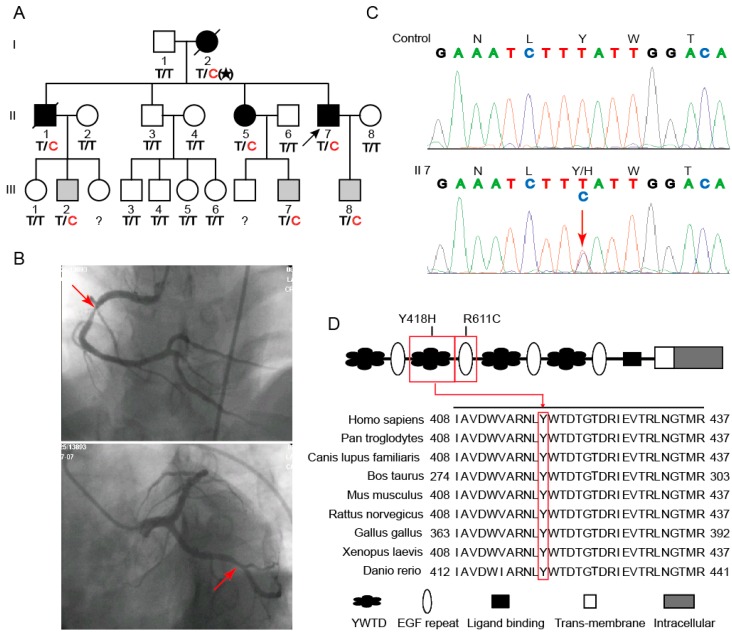
Novel mutation of *LRP6* identified in a CAD pedigree. (**A**) Pedigree of the family with the *LRP6* Y418H mutation. Numbered individuals correspond to those in Table 1. Circles represent females; Squares represent males; Proband is indicated by the arrow; Individuals with CAD are indicated by black symbols; Individuals without CAD are shown as unfilled symbols; Presymptomatic carriers are shown by symbols by gray symbols; Symbols with a slash through them indicate deceased subjects; Genotypes of the *LRP6* mutation were shown below the symbols who were willing to participate in the study; Filled stars indicates that the genotype of subject I2 was speculated; Individuals who were not available for studied are indicated with question mark; (**B**) Coronary angiogram of the proband. The red arrows indicates the stenosis; (**C**) DNA sequence analysis for a segment of *LRP6* exon 6 from a healthy control (**top**) and the proband (**below**). A red arrow points out a single base mutation in the proband, and it results in the substitution of histidine for tyrosine at codon 418; (**D**) Conservatism analysis by interspecies alignments. The mutation position is indicated with a red frame.

DNA was extracted from the peripheral blood lymphocytes of 40 probands, including 19 hyperlipidemic and 21 normolipidemic CAD patients from northern China (Table S1). After amplifying the genomic DNA with primers, as shown in Tables S2 and S3, all exons and intron-exon boundaries of *LRP6* and *MEF2A* were sequenced. As a result, a novel heterozygous variant in exon 6 of *LRP6* was identified in a proband, belonging to a patient who was 41 years old and diagnosed with myocardial infarction without hyperlipidemia. This proband (subject II7) was from a family with three generations, involving 20 patients and non-affected first-order relatives (Figure 1A). Two of his three major coronary arteries showed at least 80% stenosis (Figure 1B), leading to percutaneous coronary intervention. The variant was located in a domain named YWTD (Tyr–Trp–Thr–Asp), causing a substitution of histidine to tyrosine (Y418H) (Figure 1C). The multiple alignments using a basic local alignment search tool (BLAST) in the NCBI database showed that this region was highly conserved from *Denio rerio* to *Homo sapiens* (Figure 1D). Y418H was predicted to be a probable damaging change with a score of 0.994 in Polyphen-2 (available online: http://genetics.bwh.harvard.edu/pph2/, Table S4). We sequenced exon 6 of *LRP6* in another 1025 unrelated healthy individuals and found no variants.

In the Y418H family, another four members were previously diagnosed with CAD or suffered from sudden cardiac death before age 50 (men) or 55 (women), and two of them have been deceased. In addition to CAD, the proband (subject II7) and his sister (subject II5) were diagnosed with hypertension and diabetes. The third generation is too young and has no clinical symptoms, and is thus not subjected to clinical diagnosis. We genotyped 17 individuals from this family and found that the Y418H variant was co-segregated with CAD phenotypes (Figure 1A). It was speculated that this mutation came from subject I2. Eleven family members were available for measurement of fasting glucose, total cholesterol (TC), triglycerides, LDL-C, and HDL-C (Table 1). For subject II3 (unaffected) and subject II5 (CAD), who had not taken lipid-lowering drugs during the past six months, blood lipid levels were similar. Additionally, despite the similar ages, the blood lipid levels of Y418H-carriers III7 and III8 were significantly different. These suggested that Y418H is not co-segregated with dyslipidemia and that this familial CAD is not linked to hyperlipidemia.

Although no other mutations in *LRP6* or *MEF2A* were found in the other 39 probands, we noticed several known single nucleotide polymorphisms (SNPs) included in the single nucleotide polymorphism database (dbSNP) during screening (data not shown). Nevertheless, we did not calculate their minor allele frequencies because of the limited number of samples.

### 2.2. LRP6_Y418H_ Weakened the Wnt Signaling Pathway

**Figure 2:**
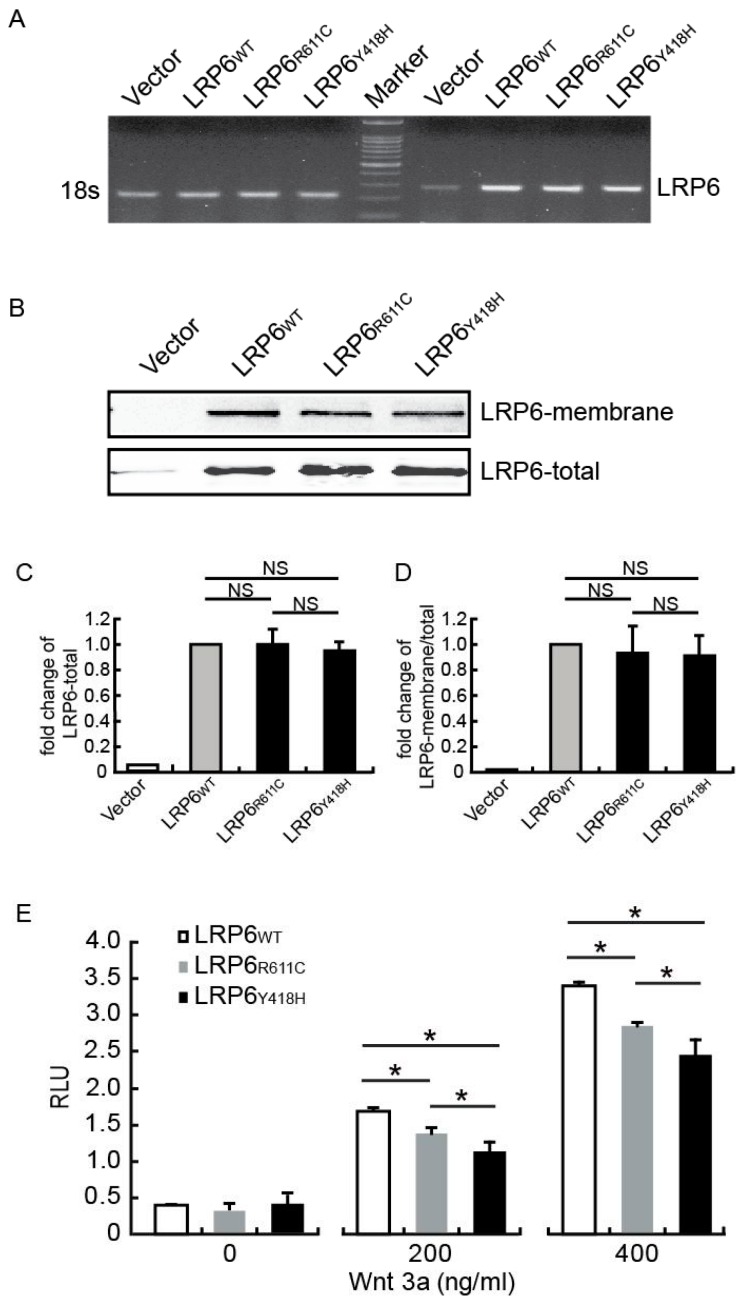
Effect of *LRP6_Y418H_* and *LRP6_R611C_* on Wnt signal transduction. (**A**) RT-PCR showed that there was no significant difference between the over-expression levels of LRP6_WT_/LRP6_R611C_/LRP6_Y418H_; (**B**) Western blot showed no significant difference between total expression levels or membrane location of wild-type and mutant LRP6. Results were replicated three times and a representative figure was shown; (**C**,**D**) Statistical result of (**B**); (**E**) Luciferase assay was performed with different amount of Wnt3a. RLU: relative light units. Results were obtained with four independent transfections. Error bars, standard deviation. * indicate *p*-value for one-way ANOVA plus post-hoc test <0.05. NS, not significant in one-way ANOVA plus post-hoc test.

As a receptor, *LRP6* activates the canonical Wnt-mediated signal pathway. To determine whether the mutation Y418H impaired Wnt signaling transduction, a comparative study was carried out among wild-type (*LRP6_WT_*), mutant *LRP6* with Y418H (*LRP6_Y418H_*) and mutant *LRP6* with a previously reported mutation R611C (*LRP6_R611C_*) [12]. We transfected human umbilical vein endothelial cells (HUVEC) with the same amount of plasmids encoding LRP6_WT_, LRP6_R611C_ or LRP6_Y418H_, which were supported by RT-PCR (Figure 2A). Western blot showed that when an equal amount of protein was loaded, the distribution of mutant LRP6 protein in the cellular membrane faction was similar to wild type (Figure 2B–D). The luciferase reporter activity indicating the extent of Wnt signal activation was then measured. We found that the Wnt3a-induced signaling was decreased 20% and 30% for *LRP6_R611C_* and *LRP6_Y418H_* compared with *LRP6_wt_*, respectively (*p* < 0.05) (Figure 2E). A more severe reduction was observed in the *LRP6_Y418H_*- than in the *LRP6_R611C_*-treated group (*p* < 0.05).

### 2.3. LRP6_Y418H_ Impaired Endothelial Cell Functions

**Figure 3:**
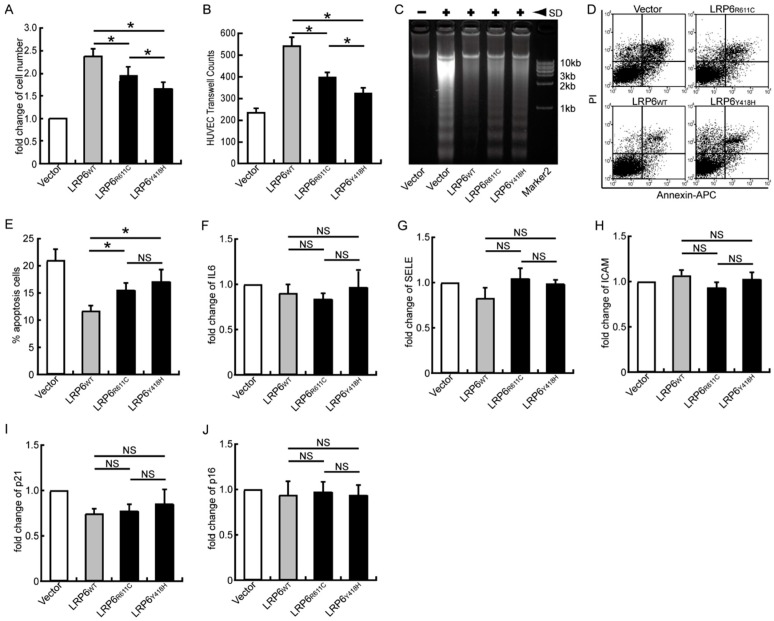
Effect of *LRP6_Y418H_* and *LRP6_R611C_* on endothelial cell functions; (**A**) Comparison of endothelial cells’ proliferation. The same amount of cells was over-expressed with wild-type LRP6 or mutant. After 48 h, cell number was counted for each group. Results were calculated using eight random fields from four independent biological replications; (**B**) Comparison of endothelial cell migration. Results were calculated using six views from three independent replications of Boyden chamber assay; (**C**) Electrophorogram for DNA ladder assay. SD, serum depletion; (**D**) Scatter diagram from flow cytometry assay. Data were presented in 2D diagrams plotting PI against Annexin-APC. Compensation for background fluorescence was performed by measuring target signals of single color controls and negative controls. Two quadrants in the right-side diagram represent apoptotic cells; (**E**) Statistical result for proportion of apoptotic cells in each group. Three biological repeats were taken into calculation; (**F**–**J**) Relative mRNA level of markers for endothelial cell activation (*IL6*, *SELE*, and *ICAM*) and senescence (*P21* and *P16*). Three biological repeats were taken into calculation. Error bars, standard deviation. * indicate *p*-value for one-way ANOVA plus post-hoc test <0.05. NS, not significant.

Endothelial cell dysfunction contributes to atherosclerogenesis. To investigate the possible mechanisms of atherosclerosis associated with *LRP6_Y418H_*, we first screened the effect of this mutation on endothelial cell functions, including proliferation, migration, and anti-apoptosis, which were critical to maintaining the integrity of the endothelium. A significantly decreased proliferation and migration was observed in HUVEC over-expressing *LRP6_R611C_* and *LRP6_Y418H_* (all *p* < 0.05), and the effect of *LRP6_Y418H_* was more profound than that of *LRP6_R611C_* (Figure 3A,B as well as Supplementary Figure S1). The over-expression of *LRP6* (wild type and mutant) protected endothelial cells from the serum withdrawal-induced apoptosis, as shown by apoptotic DNA ladders (Figure 3C) and flow cytometric analyses (Figure 3D,E); the anti-apoptotic ability, however, was decreased in the cells infected by the mutant *LRP6* (*LRP6_R611C_* and *LRP6_Y418H_*). No significant difference was observed between the two mutations.

We then assessed how the mutations influenced the inflammatory responses of endothelial cells and found no differences among the wild-type and two mutations in the mRNA expression of *IL6* (Figure 3F), *SELE* (Figure 3G), and *ICAM-1* (Figure 3H).

Finally, no differences were found in the mRNA expression of *P21* and *P16*, two markers for cell senescence, among all groups, suggesting senescence was not involved (Figure 3I,J).

### 2.4. The Influence of LRP6_Y418H_ on Cellular LDL-C Clearance

**Figure 4:**
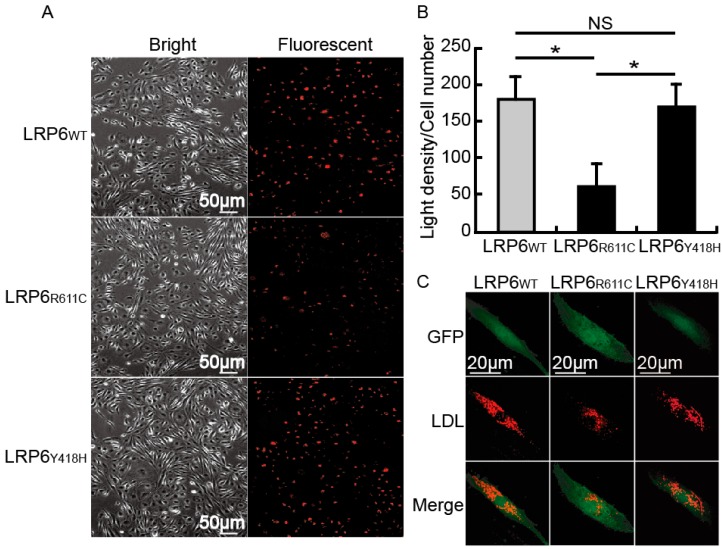
Effect of *LRP6_Y418H_* and *LRP6_R611C_* on LDL uptake in HUVEC. (**A**) An overview of the Dil-LDL uptake in HUVEC; Left, bright field; Right, Fluorescent field; (**B**) Quantitative results were calculated using six random views from three independent replications. Error bars, standard deviation. * indicate *p*-value for one-way ANOVA plus post-hoc test <0.05. NS, not significant; (**C**) Enlarged views for single cells. Top, GFP translated by IRES following *LRP6*; Middle, Dil-LDL; Bottom, merged data.

Compared with *LRP6_wt_*, *LRP6_R611C_* has been shown to decrease cellular LDL-C clearance. We examined whether *LRP6_Y418H_* had a similar effect. After being incubated with LDL-C labeled by Dil (a red dye), cells transfected with *LRP6_R611C_* presented a reduced LDL uptake compared to the *LRP6_wt_* cells (*p* < 0.05) (Figure 4A,B), but no differences existed between *LRP6_wt_* and *LRP6_Y418H_*.

## 3. Discussion

Here we report a novel heterozygous mutation of LRP6 in a Chinese normolipidemic CAD family, which leads to a substitution of histidine to tyrosine (Y418H) in an evolutionarily conserved domain, YWTD. LRP6Y418H does not alter the lipid transportation; however, it weakens the Wnt signaling pathway and exhibits deleterious effects on the proliferation, migration, and survival of endothelial cells. Our study suggests that impaired endothelial functions caused by genetic mutation are critical in the pathogenesis of normolipidemic CAD.

Over the past decades, a large number of genetic studies have been performed to search for the genes or loci associated with CAD mostly with hyperlipidemia, but genetic knowledge on normolipidemic CAD remains limited [20]. Here, we identified a genetic variant (Y418H) of LRP6 in a Chinese normolipidemic CAD family and provided several lines of evidence to show that this genetic variant was a possible defect for familial normolipidemic CAD. Genetically, Y418H occurred in an evolutionarily conserved YWTD domain, was co-segregated with CAD phenotypes in the family, and was not detected in 1025 healthy individuals in this study. In addition, we inspected this mutation in three other databases. Y418H was not found in the NHLBI GO Exome Sequencing Project (ESP) (6503 samples) and in the in-house control test (221 samples); however, there were two carriers in 1000GP (2504 samples), yielding an allele frequency of 0.0004 (Table S4). Due to the lack of diagnosis, we cannot rule out whether or not those carries are potential CAD patients occurring in Han Chinese South (CHS). It is very interesting to note that we have observed LRP6 mutations in sporadic Chinese CAD patients [21]. With the notion from the present study that *LRP6* can be a candidate gene for normolipidemic CAD, we checked the blood lipid levels and found that they were not elevated in their first clinic visits (Table S5). Differently, mutations in *LRP6* were screened out in American kindreds with early-onset hyperlipidemic CAD [22]. With the limited numbers of mutations found by far, it is difficult to make solid correlations between race, genotypes (positions of the mutations in *LRP6* gene) and phenotypes (normolipidemic or hyperlipidemic CAD). It appears clear that *LRP6* is a plausible candidate gene for both normolipidemic and hyperlipidemic CAD.

Functionally, Y418H is predicted to be a damaging allele with a Polyphen2 score of 0.994 that is higher than that of R611C. We demonstrated that LRP6Y418H weakened the ability in proliferation, migration, and survival of endothelial cells when exposed to stress. It is known that homeostasis or prompt renewal of endothelial cells is critical to maintain the integrity of the endothelium. Denuded by mechanical injuries, the endothelium can be amended by adjacent endothelial cells and endothelial progenitor cells as well [3,23,24,25,26,27,28,29,30,31]. This suggests that, in addition to endothelial progenitor cells, the survival, proliferation, and migration of endothelial cells localized in the zone adjacent to the injured region are important to maintain the homeostasis. The impaired or dysfunctional endothelium associated with LRP6Y418H should increase the susceptibility, together with other risk factors, such as hypertension and diabetes in the affected individuals, to develop atherosclerosis in coronary arteries. Finally, we showed that LRP6Y418H weakened the Wnt signaling pathway that had been reported to contribute to familial CAD [12]. The extracellular structure of the LRP6 protein is mainly composed of four beta propellers (BP) that contain six YWTD repeats which are separated by four EGF-like domains. It was reported that the four BPs have different functions, for example BP1 and BP2 are mainly responsible for the binding of Wnt and Wise [32,33]. Strikingly, most mutations in Supplementary Table S4 that were predicted to be seriously functionally damaged reside in the second propeller and EGF-like domain, suggesting the importance of the second YWTD-EGF structure and Wnt signal for CAD. This genetic and functional evidence supports that *LRP6* is a reasonable candidate gene for normolipidemic familial CAD.

Since R611C and Y418H were found in two CAD families, we compared clinical phenotypes and functions of the two mutant LRP6s. In clinical phenotypes, two families had some overlapping phenotypes: (1) some individuals in the families suffered from sudden cardiac death; and (2) the affected subjects (after age 40) had multiple CAD risk factors such as hypertension and diabetes. However, the blood lipid was dramatically different between the CAD patients in the two families: the Y418H family was normolipidemic while the R611C family was hyperlipidemic; high levels of LDL-C and triglycerides were co-segregated with CAD in the R611C family. Functionally distinct from Y418H, the R611C mutation was in the EGF-like domain and impaired the cellular LDL-C uptake. This was in agreement with a recent finding that the R611C mutation resulted in decreased LDL-C clearance [34] and reduced LRP6 activity in LRP6R611C mice that had elevated plasma LDL-C and TG levels and fatty liver [35]. Nonetheless, two mutations (Y418H and R611C) at the different positions of the *LRP6* gene can be linked to normolipidemic and hyperlipidemic CAD, two subtypes of familial CAD, suggestive of the importance of LRP6.

LRP6, a trans-membrane protein of the low-density lipoprotein receptor (LDLR) family, was identified based on its homology with the LDLR gene [36]. As a receptor of the canonical Wnt signaling pathway, LRP6, together with LRP5 and Frizzled, activates β-catenin-TCF/LEF, and regulates downstream signaling which is important in the development and maintenance of the cardiovascular system [37]. For example, activation of this pathway induces the proliferation, migration, and survival of endothelial cells [38,39,40]. We in this study demonstrated that LRP6Y418H attenuated the Wnt3a-activated signaling pathway, decreased proliferation and mobility, and weakened the anti-apoptotic response of endothelial cells. Similarly, it has been shown that mutations of LRP6 found in sporadic CAD patients attenuated proliferation and migration of HUVEC as well as the Wnt signal [21]. LRP6R611C also attenuated the Wnt3a-activated signaling pathway [12]. These suggest that mutant LRP6 impairs endothelial cell functions, possibly through the attenuation of the Wnt-signaling pathway. However, how other risk factors, such as hypertension and diabetes, interact with the genetic defect in atherosclerogenesis needs to be further investigated.

Several limitations exist in this study: (1) the third generation of the studied pedigree is still young, and we cannot claim whether or not the mutation carriers in this generation are CAD patients; (2) LRP6 has a broad range of functions, and whether other signal pathways are involved in the pathogenic mechanism of this family cannot be excluded. For example, LRP6 is a co-receptor for multiple fibrogenic signaling pathways in pericytes and myofibroblasts [41]. It is not clear whether or not these pathways contribute to CAD associated with the LRP6 mutations; and (3) although Wang et al. identified *MEF2A* as a causal gene for CAD, we did not find any mutation in this gene during our first screening. Similar to our finding, Lieb et al. suggested the lack of association between the *MEF2A* gene and myocardial infarction [42]. However, limited by the modest sample number, we cannot rule out the role of *MEF2A* in CAD.

In conclusion, we identified a mutation (Y418H) in the YWTD domain of LRP6 that co-segregated with normolipidemic CAD in a Han Chinese family that impaired endothelial cell functions, implying that endothelial dysfunctions associated with a genetic mutation play an important role in normolipidemic CAD.

## 4. Materials and Methods

### 4.1. Study Subjects

We recruited 40 Chinese Han families with CAD that were ascertained through probands and included more than two early-onset CAD/ myocardial infarction (MI) patients (less than 50 years old for males or 55 years old for females). Blood pressure, glucose, hypertension, and diabetes mellitus were measured or defined [43]. Hyperlipidemia or normolipidemia were defined based on clinical diagnosis. 1025 unrelated Han Chinese controls were general healthy donors [44]. Written informed consent was obtained from all participants. The study was conducted in agreement with the principles outlined in the Declaration of Helsinki and approved by the Institutional Review Board, Institute of Molecular Medicine at Peking University.

### 4.2. Cell Culture

Primary human umbilical vein endothelial cells (HUVECs) were isolated from fresh human umbilical veins, cultured in complete Endothelial Cell Medium (ECM, ScienCell), and maintained at 37 °C in 5% CO_2_. The informed consents were signed by babies’ fathers. The study was conducted in agreement with the principles outlined in the Declaration of Helsinki and approved by the Institutional Review Board, Institute of Molecular Medicine at Peking University.

### 4.3. Mutational Screening

Human genomic DNA was isolated as described previously [44]. The exons of the target genes and their flanking exon-intron boundaries were amplified by PCR using specific primers listed in Tables S2 and S3. The amplified DNA fragments were purified and subjected to direct sequencing on ABI 3130XL according to the manual description of BigDye v3.1 kit.

### 4.4. Wnt Signaling Analysis

cDNA clone of LRP6 (pCR-XL-TOPO-LRP6) was purchased from FulenGen. cDNA sequence was validated by sequencing and inserted into pShuttle-IRES-hrGFP-1 vector. Mutations (Y418H and R611C, a previously reported mutation [12]) were introduced by PCR-based mutagenesis, respectively. The mutations were verified by DNA sequencing. HUVEC was electric transfected with equal amount of plasmids expressing wild-type (LRP6wt) and mutant LRP6 (LRP6R611C or LRP6Y418H), respectively. A Wnt pathway reporter system was utilized as previously described [12]. Briefly, plasmids encoding wild-type LRP6 or mutants, LEF-1, firefly luciferase, and renilla luciferase were introduced into cells by transfection. After 24 h, cells were lysed and luciferase reporter activity indicating the extent of Wnt signal activation was measured in accordance with the dual luciferase assay specifications (Promega, Hollow Road Madison, WI, USA).

### 4.5. Quantitative Real-Time PCR

Total RNA was extracted using Trizol reagent (Invitrogen, Waltham, MA, USA). Reverse transcription and quantitative real-time PCR were performed as previously described [45]. Information of specific primers is listed in Table S6. Results were normalized to 18S rRNA.

### 4.6. Western Blot Analysis of LRP6

Total and membrane protein were extracted using kit from Beyotime Biotechnology. Western blot was performed to detect the wild type and mutant LRP6 protein (primary antibody for LRP6: sc-25317, Santa Cruz, Dallas, TX, USA) based on the protocol previously reported [46]. GAPDH was used as a loading control.

### 4.7. HUVEC Proliferation, Migration, and Apoptosis Analysis

For overexpression wild-type LRP6 or mutants in HUVECs, infection was performed with adenovirus that constructed following manufacturer’s protocol for AdEasy system (Stratagene, Santa Clara, CA, USA) [47]. We controlled the same over-expressional level among different groups by adjusting the amount of virus added. Proliferation was evaluated using MTT (Sigma, St. Louis, MO, USA) assay and actual cell number count, and migration was evaluated Boyden Chamber assay [48]. To avoiding the effect of proliferation, we controlled the migration time within 12 h. Apoptosis was presented by both DNA ladder gel electrophoresis [49] and FACS analysis (BD Biosciences Clontech Kit, San Jose, CA, USA).

### 4.8. Dil-LDL Uptake

HUVECs were placed in a six-well plate containing 20 μg/dL cholesterol and 2 μg/mL 25-hydroxycholesterol to down-regulate the endogenous LDLR (Supplementary Figure S2). After 24 h, 10 μg/mL Dil-LDL was added and incubated at 37 °C for six hours. Cells were washed three times with PBS containing 1% FBS and were fixed in 4% paraformaldehyde. Specimens were then examined by confocal microscope.

### 4.9. Statistic Analysis

Results are expressed as mean ± SD as indicated in the legends for measurement data. Comparisons were performed using one-way ANOVA with post-hoc in SPSS software. *p* < 0.05 was considered statistically significant.

## Figures and Tables

**Table 1:** Clinical characteristic of members with CAD pedigree

ID	Age/Onset Age	Gender	BMI	SBP (mmHg)	DBP (mmHg)	Anti-HT Drug	Glucose mmol/L 3.9~6.1	Anti-D Drug	Triglyceride mmol/L 0.56~1.8	TC mmol/L 2.9~6.0	HDL-C mmol/L 0.8~1.8	LDL-C mmol/L 1.6~3.2	Anti-HL Drug	CAD Status
I1	86	male	23.9	140	80	no	NA	no	NA	NA	NA	NA	no	Unaffected
I2 *	65 ^†^/NA	female	NA	NA	NA	NA	NA	NA	NA	NA	NA	NA	NA	CAD/Stroke
II1 *	49 ^†^/49	male	NA	NA	NA	NA	NA	NA	NA	NA	NA	NA	NA	SCD
II3	58	male	25.0	140	80	no	6.06	no	1.80	5.46	1.31	3.53	no	Unaffected
II5 *	47/46	female	27.9	170	100	yes	8.08	yes	1.35	6.93	2.10	3.76	no	CAD
II7 *	44/41	male	25.4	135	80	yes	7.16	no	1.57	3.23	1.02	1.76	yes	CAD
III1	33	female	22.2	115	75	no	4.58	no	0.56	4.51	1.68	2.18	no	Unknown
III2 *	31	male	24.9	130	80	no	4.69	no	2.24	6.24	1.18	3.66	no	Unknown
III3	34	male	23.7	150	100	no	4.53	no	1.24	4.87	1.12	3.31	no	Unknown
III4	28	male	24.2	140	80	no	3.83	no	1.26	4.93	1.65	2.32	no	Unknown
III5	35	female	23.4	120	80	no	4.54	no	0.68	4.59	1.62	2.27	no	Unknown
III6	30	female	20.3	105	70	no	3.42	no	0.94	3.97	1.38	2.12	no	Unknown
III7 *	22	male	21.2	110	70	no	4.76	no	1.18	3.60	1.23	1.77	no	Unknown
III8 *	20	male	30.1	130	85	no	4.58	no	1.29	6.12	1.48	3.81	no	Unknown

ID corresponds to those in Figure 1; BMI: body mass index; SBP, systolic blood pressure; DBP: diastolic blood pressure; anti-H: anti-hypertension; anti-D: anti-diabetes; anti-HL: anti-hyperlipidemia; TC: total cholesterol; HDL-C: high density lipoprotein cholesterol; LDL-C: low density lipoprotein cholesterol; *: Y418H carrier; ^†^: deceased; SCD: sudden cardiac death; NA: not available.

## Supplementary Materials

Supplementary materials can be found at http://www.mdpi.com/1422-0067/17/7/1173/s1.
