# Supplementary material for: Mutant LRP6 Impairs Endothelial Cell Functions Associated with Familial Normolipidemic Coronary Artery Disease
Source: Int J Mol Sci. 2016 Jul 22;17(7):1173. doi: 10.3390/ijms17071173 (PMC4964544; doi:10.3390/ijms17071173)
Supplement: Supplementary file 1 [file ijms-17-01173-s001.pdf]

Supplementary Materials: Mutant *LRP6* Impairs Endothelial Cell Functions Associated with Familial Normolipidemic Coronary Artery Disease

Jian Guo, Yang Li, Yi-Hong Ren, Zhijun Sun, Jie Dong, Han Yan, Yujun Xu, Dao Wen Wang, Gu-Yan Zheng, Jie Du and Xiao-Li Tian

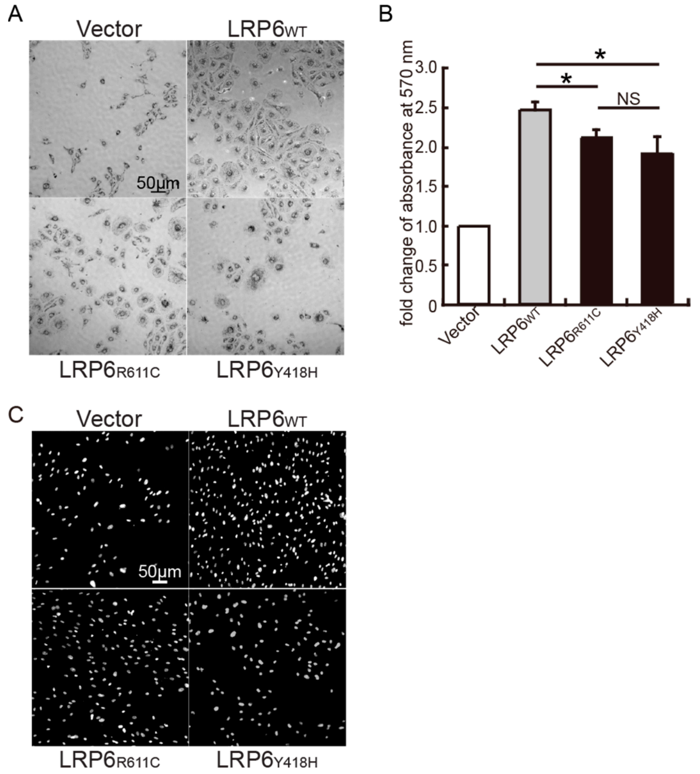

Figure S1. Supplementary results of cell proliferation and migration. (**A**) Pictures taken before DMSO was added during MTT assay; (**B**) Statistical result for MTT assay and calculated with three independent replications. (*) indicates p-value for one-way ANOVA plus post-hoc test <0.05. NS, not significant; (**C**) Pictures used for cell counting in Boyden chamber assay.

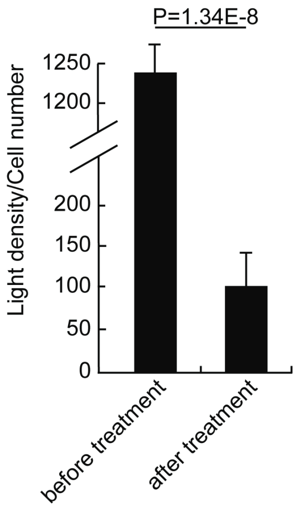

Figure S2. LDL uptake of HUVEC before and after endogenous LDLR was inhibited.

Table S1. Characteristics of 40 probands.

No.	Gender	Age	CAD Onset Age	BMI	HT	DM	HL	Smoke
1	male	29	29	26.88	0	0	1	0
2	male	32	31	25.90	0	0	0	0
3	male	31	31	25.71	0	0	0	1
4	male	35	34	24.44	0	0	1	1
5	male	34	34	23.66	0	0	1	1
6	male	35	35	21.72	1	0	0	0
7	male	35	35	24.82	0	0	0	1
8	male	38	38	23.18	0	1	1	1
9	male	41	40	32.03	1	0	1	0
10	male	41	40	27.38	1	0	0	1
11	male	40	40	34.94	1	0	1	1
12	male	40	40	25.31	1	0	0	0
13	female	46	40	29.09	1	1	1	0
14	male	44	41	26.78	1	0	1	1
15	male	41	41	27.94	1	1	0	1
16	male	43	42	27.05	0	0	0	1
17	male	42	42	33.77	0	0	0	1
18	male	42	42	33.03	0	0	0	1
19	male	44	43	25.61	1	0	0	1
20	male	45	44	25.21	1	0	1	1
21	male	44	44	31.49	0	1	1	1
22	male	44	44	25.56	1	0	1	0
23	male	44	44	24.39	0	0	1	1
24	female	46	44	23.05	1	0	1	0
25	male	45	45	29.05	0	0	0	1
26	male	45	45	27.34	0	1	1	0
27	male	46	45	24.00	1	0	0	1
28	male	46	45	29.76	1	0	0	1
29	male	47	45	25.97	1	1	0	1
30	male	46	46	22.23	0	0	1	1
31	female	46	46	28.51	0	0	1	0
32	male	46	46	24.16	0	0	0	1
33	male	46	46	22.99	0	1	0	1
34	male	46	46	26.30	1	0	1	1
35	male	47	46	28.73	1	0	0	0
36	female	47	46	23.83	0	0	0	0
37	male	47	47	26.85	1	1	1	0
38	female	47	47	22.94	1	0	0	0
39	male	49	47	27.78	0	0	0	1
40	male	49	47	31.05	0	1	1	1

HT, hypertension; DM, diabetes mellitus; HL, hyperlipidemia; 1, yes, 0, no.

Table S2. Primer pairs used to amplify segments of *LRP6*.

Primer	Forward (5′–3′)	Reverse (5′–3′)
EXON1	GAAGTATGAGCGTGCAGCC	CACATACAACAAGGCCACCT
EXON2-1	CAGTTTATAAGAACCAATACGT	CTCCAAGCCAATCACATGCCA
EXON2-2	CGGTGGACTTTGTGTTTAGT	GTCAGGGTGGTGTATGTCAGT
EXON3	GATATCTGCTCTGTCATGGCTT	GTATCAAGGCTTCATCGAGT
EXON4	GGAGAGGTGACGTTATGATAG	CATTAGAGTGCTACAAAGCTG
EXON5	GGCTTATCACAGTTGTTCATGT	CTCCCAAAGCAGTATAACCT
EXON6-1	CCTCTGGGTTTCAAAGTATTCT	CCACAGCAATACCATCAGGAT
EXON6-2	GAAGGCTACATCTACTGGACT	GTCAATGTTTTCAGGAACCTGTG
EXON7	GTGTAATAGGAGGGATGGATCT	CTCAAATCATTATACTGTCGACTC
EXON8	CAGGCAGTTGGATTTGTTAT	CACTGCTGACTATCTCCATCTT
EXON9	CAATGTGTGTGTCCTTCTGTGC	CTCCCTTTTAGTCCCTAGCT
EXON10	GGTTTAGACTTATGCCATATGAC	CTGTAACTATGCCATGTTCC
EXON11	GTTACTTTGCATGAATATGACTC	CAATGATAAATAGCAGCCACTG
EXON12-1	GCCAGCAAGACACTTGTATT	CAAGCAGAGGTGGGAGCAGT
EXON12-2	GCACCATCATTCAGGGCCAT	CTTTCTCTCATTCTAGCTTGCT
EXON13	GGATGATAGAGGATGTAGGGT	CAGAGTGGTTGGTGAGTCC
EXON14	GGTGTAAGCATTGTGACTGATT	GAGGAGAGTCTCAGAAGCCA
EXON15	CTCTACCTCCATTTGTCTTAAT	GCAAGAAAAACACAATAGATGAAT
EXON16	GTATAACTTGACCCACATGAGT	CACATGATTCCTCCAATTAGCT
EXON17	CTCTTCTCTCCCACTTCTCTCC	GGAATATGTTTGCATTTAGGGT
EXON18	GTACTTCTGTGTGACCATGATTG	GGATTGCATGCTGAGGCACT
EXON19	GCCAGAGTTTGACTTTACATG	CTCGATAAGTAAACCTCACACAT
EXON20	CACATCCTATTAGCAAGCCCT	GCAGTCTTGCAGGACCAAAG
EXON21	CCTACTGTTGAATGAAATTGCTT	GTGGTAAGTCCTTTGAGCCT
EXON22	CTGTTTGAGGTCAATTCTTTGGA	GGCTATATCAGGTCCACAACT
EXON23-1	GTAGTTGACATCTAATACCTCT	CTCCTCTGCTGACAAGTATT
EXON23-2	CCAAGGGCTATACCAGTGAC	CCCTCCAGATCTCAACCAAAT

Table S3. Primer pairs used to amplify segments of *MEF2A*.

Primer	Forward (5′–3′)	Reverse (5′–3′)
EXON1	GAGAAGCGAGGAAGGGAAC	CCTACTCGGTCCCCTAAGCG
EXON2	CTGCTAGGAATGGTGTTGAGT	ATAACTGCCATTCAATCTCAC
EXON3	TTGTATAACACTTACATGATTTGT	CCAACCATTCTGTCCTATGT
EXON4	AGATTCATCTTCAGATAGCCCAT	ACAAGTCATTCTGACAGTTAATGC
EXON5	AGTTCATTCCGTCTGTGCTCTCT	GCAACAAGATGTTTGGTCAATCTCT
EXON6	AGTAACTTGAGTTACCTTGCC	GTATCAGTGATTCTTGCTCATC
EXON7	TCTCTATTCAGTTCACGTTCAGTT	TGTATTAGTGAAAGTACCCTTCAG
EXON8	GATACTCAAACCTGTAGTGAGT	GGAAGCTACAGATTGACTATGT
EXON9	TGTGAGTACCAACAGTCTTAGT	GGTTAGATAACAACACGTAAGAG
EXON10	TCACATCATCAGTGCTTCAGAA	CACAGAAGCACACGTTGATCA
EXON11	ATAGATTCCGTATGGACCTTCC	AAGAGAGTGTGTAGGCCAGGAGT
EXON12	GCAGAGGTACTTGCAAGCCAT	GATATGTAGGGCAGGTCACTG

Table S4. Summary of *LRP6* mutations found in CAD patients. MAF, minor allele frequency; 1000GP, 1000 genome project; CDX, the Dai nationality in China；CHB, northern China；CHS, southern China；KHV, Vietnam; JPT, Japan; ESP, NHLBI GO Exome Sequencing Project; RefInAll, reference allele in all sample; NA, not available; PhastCons scores represent the degree of conservation of a nucleotide position; GERP: Genomic Evolutionary Rate Profiling, a method for producing position-specific estimates of evolutionary constraint using maximum likelihood evolutionary rate estimation. PolyPhen, polymorphism phenotyping that predicts the possible effect of an amino acid substitution on the structure and function; SIFT, predicts whether an amino acid substitution affects protein function based on the degree of conservation of amino acid residues in sequence alignments derived from closely related sequences, collected through PSI-BLAST (T: tolerated, D: damaging); NL-CAD, normolipidemic CAD; HL-CAD, hyperlipidemic CAD.

Chr.Pos (GRCh37/hg19)	Mutant_ Protein	Mutant_ mRNA	Exon	Functional Domain	dbSNP-144	1000GP (Phase III) (MAF)	ESP (MAF)	In House Database (MAF)	Phast Cons	GERP	Poly Phen2	SIFT	Pheno-Type	Ref.
chr12:12397399	K82N	c.388 A>T	2	/	rs199693693	0.0028	RefInAll	0.0023	1	4.43	0.021	T	NL-CAD	[21]
chr12:12334271	R360H	c.1221 C>T	5	Second β propellers	rs141212743	RefInAll	0.00015	NA	1	5.82	0.915	D	HL-CAD	[22]
chr12:12334052	N433S	c.1440 T>C	6	Second β propellers	rs397515473	RefInAll	RefInAll	RefInAll	1	5.62	0.917	D	HL-CAD	[22]
chr12:12334098	Y418H	c.1394 A>G	6	Second β propellers	rs200373003	0.00040	RefInAll	RefInAll	1	5.72	0.994	D	NL-CAD	This study
chr12:12332871	R473Q	c.1560 C>T	7	Second β propellers	rs397515474	RefInAll	RefInAll	RefInAll	1	5.82	0.897	T	HL-CAD	[22]
chr12:12332826	S488Y	c.1605 C>A	7	Second β propellers	NA	RefInAll	RefInAll	RefInAll	1	5.82	0.117	D	NL-CAD	[21]
chr12:12317428	R611C	c.1973 G>A	9	Second EGF-like	rs121918313	RefInAll	RefInAll	RefInAll	1	5.65	0.586	D	HL-CAD	[12]
chr12:12301886	P1066T	c.3338 C>A	14	/	NA	RefInAll	RefInAll	RefInAll	1	4.82	0.917	D	NL-CAD	[21]
chr12:12288225	P1206H	c.3759 C>A	17	/	rs764130366	RefInAll	RefInAll	RefInAll	1	5.56	0.975	T	NL-CAD	[21]
chr12:12284935	I1264V	c.3932 A>G	18	LDLA	rs549373552	0.00020	RefInAll	RefInAll	1	5.92	0.877	D	NL-CAD	[21]

Table S5. Clinical characteristics of sporadic mutation carriers.

Individual ID	Mutation	Domain	Sex	Onset Age	TG (mmol/L)	TC (mmol/L)	HDL-C (mmol/L)	LDL-C (mmol/L)
DP0539	*K82N*	/	male	46	4.34	4.69	0.72	2.08
DP0703	*K82N*	/	male	52	3.90	4.43	0.84	2.48
DP0426	*S488Y*	YWTD	male	51	1.62	6.01	0.96	4.07
DP0007	*P1066T*	/	male	39	1.18	4.14	1.12	2.20
DP0968	*P1066T*	/	male	55	1.62	3.41	1.06	1.63
DP0999	*P1206H*	/	male	55	2.50	4.71	1.02	3.04
DP1201	*P1206H*	/	male	54	0.64	4.43	0.97	3.24
DP0578	*I1264V*	LDLA	male	45	0.41	3.70	1.08	2.06

TG, triglyceride; TC, total cholesterol; HDL-C, high-density lipoprotein cholesterol; LDL-C, low-density lipoprotein cholesterol.

Table S6. Primer pairs used to amplify markers of endothelial cell activation.

Primer	Forward (5′–3′)	Reverse (5′–3′)
IL-6	GTACATCCTCGACGGCATCT	CATCTTTGGAAGGTTCAGGTT
SELE	GGACACAGCAAATCCCAGTT	CTGCCAGAAGCACTAGGAAG
ICAM	CTGACGTGTGCAGTAATACT	GGCTTCGTCAGAATCACGT
